# Flow Behavior of Nanoparticle Agglomerates in a Fluidized Bed Simulated with Porous-Structure-Based Drag Laws

**DOI:** 10.3390/nano14121057

**Published:** 2024-06-19

**Authors:** Shaowei Wang, Xiaobing Hu, Niannian Liu, Huanpeng Liu

**Affiliations:** 1 Energy and Power Engineering Institute, Henan University of Science and Technology, Luoyang 471003, China; 2 Department of Engineering Mathematics, University of Bristol, Bristol BS8 1QU, UK; 3 School of Energy Science and Engineering, Harbin Institute of Technology, Harbin 150006, China; liuhuanpeng@hit.edu.cn

**Keywords:** nanoparticle agglomerate, fluidization, flow behavior, drag laws, simulation

## Abstract

Fluidization bed reactor is an attractive method to synthesize and process quantities of functional nanoparticles, due to the large gas–solid contact area and its potential scalability. Nanoparticles fluidize not individually but as a form of porous agglomerates with a typical porosity above 90%. The porous structure has a significant effect on the hydrodynamic behavior of a single nanoparticle agglomerate, but its influence on the flow behavior of nanoparticle agglomerates in a fluidized bed is currently unclear. In the present study, a drag model was developed to consider the porous structure effects of nanoparticle agglomerates by incorporating porous-structure-based drag laws in the Eulerian–Eulerian two-fluid model. Numerical simulations were performed from particulate to bubbling fluidization state to evaluate the applicability of porous-structure-based drag laws. Results obtained for the minimum fluidization and bubbling velocities, bed expansion ratio, and agglomerate dispersion coefficient show that, compared with the drag law of solid sphere, the porous-structure-based drag laws, especially the drag law of fractal porous spheres, provide a closer fit to the experimental data. This indicates that the pore structures have a great impact on gas–solid flow behavior of nanoparticle agglomerates, and the porous-structure-based drag laws are more suitable for describing flows in nanoparticle agglomerate fluidized beds.

## 1. Introduction

Due to their unique physical and chemical properties, nanoparticles have aroused great interests with the rise of nanotechnology over the past few decades [1]. They have great potential in the applications of energy storage [2], medical [3], catalytic [4,5], photo luminescent [6] and sensing fields [7]. Fluidization is an attractive method to process quantities of nanoparticles because of the large gas–solid contact area and its potential scalability [8,9]. Peculiarly, nanoparticles do not fluidize individually but form agglomerates as a result of strong inter-particle forces [10]. The design and scale-up of fluidized beds require understanding of the group flow behavior of the nanoparticle agglomerates.

Computational fluid dynamics is an effective tool for understanding the hydrodynamic characteristics of gas–solid flows in fluidized beds [11,12]. The Eulerian–Eulerian model has been widely used to study the effects of particle–particle and gas–particle interactions on the hydrodynamics of fluidized beds containing particles from Geldart group A to D [13]. The drag force between the gas and particles is one of the dominant hydrodynamic forces in a fluidized bed. Due to the very fragile nature of the fluidized nanoparticle agglomerates, there is no drag law yet suitable for them proposed experimentally in the literature. Some previous studies have assumed fluidized nanoparticle agglomerates as solid spheres and used the drag law of solid sphere particles for hydrodynamic analysis instead [14]. It has been reported that the Eulerian—Eulerian approach with the drag law of solid sphere particles is inaccurate for predicting the hydrodynamics of Geldart A particles in certain cases [15]. The fluidized nanoparticle agglomerates have similar porous structures to clusters formed by Geldart A particles in a fluidized bed but with much higher porosity. The method of treating fluidized nanoparticle agglomerates as solid spheres ignores the extremely porous nature of fluidized nanoparticle agglomerates, which is not consistent with the actual situation and may bring some simulation error. Therefore, the sensibility for modeling the fluidization of nanoparticle agglomerates with drag laws of solid sphere particles under different flow regimes needs to be further assessed.

It is widely accepted that the fluidized nanoparticle agglomerates are highly porous structures with a typical porosity of 90% or higher [16]. This structure is formed by primary nanoparticles in a hierarchical form and possesses fractal properties [17]. Previous numerical studies of the hydrodynamics of a single fluidized nanoparticle agglomerate show that, because of the internal flow through these highly porous structures, the hydrodynamics of a single fluidized nanoparticle agglomerate are different from the impermeable mediums [18]. A single nanoparticle agglomerate experiences much lower drag force compared with the equivalent solid sphere particle with the same size and density under the fluid flow at the same Reynolds number. By considering its structural characteristics, some drag laws of a single nanoparticle agglomerate have been proposed by treating it as a homogenous porous sphere particle or a fractal porous sphere particle. It is also inferred that the porous structure of nanoparticle agglomerates may strongly affect their group behaviors during fluidization, but no study has been reported to assess the applicability of these drag laws in the fluidized bed. Therefore, the applicability for modeling the fluidization of nanoparticle agglomerates under different flow regimes with porous-structure-based drag laws needs to be evaluated.

In this regard, the fluidization behavior of nanoparticle SiO_2_ agglomerates is simulated in this work in the framework of the Eulerian–Eulerian gas–solid two-fluid model with the solid sphere drag law and the porous-structure-based drag laws. The hydrodynamic behaviors, including the pressure drop, the bed expansion ratio, and the solid mixing behavior in a fluidized bed for different fluidization velocities, are studied. The validity of different drag laws is assessed through comparisons of simulation results with the experimental data.

## 2. Materials and Methods

### 2.1. Basic Equations

The Eulerian–Eulerian two-fluid model was employed to compute gas–solid flow for nanoparticle agglomerate fluidization in this work. This method is one of the current development trends of the numerical research of gas–solid fluidization, and it has been validated as a successful method to reflect the actual gas–solid fluidization behavior in many previous research studies [19,20]. Detailed description of this model can be found in [20]. Detailed equations can be found in the Supporting Information (Sections S1 and S2). A second-order upwind method was used in the calculations. Moreover, the SIMPLE algorithm was adopted to couple pressure and velocity terms. The established model was simulated by the ANSYS Fluent 2020 R2 software. The time step is set to 0.001 s, and its convergence criterion is 1 × 10^−5^.

### 2.2. Drag Model and Drag Laws

The Huilin–Gidaspow drag model [21] was employed to couple the momentum transfer between gas and particle phases. It is a combination of the previous drag models proposed by Ergun [22] and Wen and Yu [23] by introducing a transition function to smoothly switch the drag coefficients. This model has been well used in the simulation of nanoparticle agglomerate fluidization [19,24]. As shown in Section S3 in the Supplementary Materials, the pressure drop due to friction between gas and particles is described by the Ergun equation as the bed porosity being less than 0.8 and the Wen and Yu equation as the bed porosity being larger than 0.8. It should be noted that the drag law of solid sphere particles is applied in the Huilin–Gidaspow drag model. Not only that, the drag law of the solid sphere particles is applied in all the commonly used drag models in the two-fluid framework. The adoption of these drag models means that the fluidization of nanoparticle agglomerates is simulated with a hypothesis by treating them as solid particles. However, it is widely reported that the nanoparticles are fluidized in the form of highly porous agglomerates. That porous structure may result in a reduction in the drag force. By now, only Liu et al. [25] consider the reduction in the drag force in the simulation of nanoparticle agglomerate fluidization with a drag force scale factor. This is an approach learned from simulation studies of Geldart A particles to take into account of the cluster effects [26,27]. The method has been proved to be effective in the simulation of nanoparticle agglomerate fluidization, which also shows that the effect of pore structure of nanoparticle agglomerates should be considered. However, the scale factor is of fixed value for different fluidization conditions, and it is estimated by computational experience. A new drag law considering the effects on the drag force of the agglomerate structure and the fluidization condition is necessary.

One approach considering the high porosity characteristics of nanoparticle agglomerates is treating them as homogeneous porous spheres and simulating the two-phase interactions of them through equations of porous media. This is a method often used to simulate the flow past porous particles of several hundred micrometers or larger size formed by a large number of primary particles. For example, Wu et al. [28] assumed the floc of about a few hundred micrometers as a homogeneous porous sphere and evaluated the drag force as *Re* ≤ 40. Chung et al. [29] studied the drag force of homogeneous porous sphere groups in Newtonian fluids under *Re* = 0.1 and 40 conditions. Jain and Basu [30] studied the hydrodynamic behavior of a homogeneous porous sphere with porosity between 0.70~0.99 and in the Reynolds number range of 0.01~1000. Both the porosity and Reynolds number range are suitable for nanoparticle agglomerates and the flow condition in a fluidized bed. The correlation in Equation (1) was proposed based on their results. It can be seen that the drag coefficient is a function of both the Reynolds number and the permeability. The permeability can be obtained by the Carmon-Kozeny equation in Equation (2) [30].

(1) $$C_{d_{a}}=\left[34-134\left(\frac{k}{{d_{a}}^{2}}\right)+208{\left(\frac{k}{{d_{a}}^{2}}\right)}^{2}-104{\left(\frac{k}{{d_{a}}^{2}}\right)}^{3}\right]Re^{-0.8},$$

(2) $$K=\frac{\varepsilon^{3}d_{p}^{2}}{180{\left(1-\varepsilon \right)}^{2}},$$

where $\;C_{d_{a}}$ is drag coefficient, and ε and *k* is the porosity and permeability of the porous sphere, respectively. *d_p_* and *d_a_* is the diameter of the primary particle and porous sphere, respectively.

According to Equation (1), the drag coefficient of the nanoparticle agglomerates with a size of 416 µm and a density of 36.6 kg/m^3^ formed from SiO_2_ nanoparticles with the primary particle size of 16 nm is expressed as follows:

(3) $$C_{d}=20.74Re_{s}^{-0.8}.$$

Moreover, it is also widely reported that the fluidized nanoparticle agglomerate possesses fractal property [31]. Many experimental observations also reveal that the distribution of pore structure inside the fluidized nanoparticle agglomerate is not uniform [32]. A physical model of a fractal porous sphere with continuously radially varying permeability was established for fractal nanoparticle agglomerates based on rigid deduction from fractal theory. Detailed description of this model can be found in our previous studies [18,33]. Based on the assumption of fractal porous sphere, the hydrodynamic behaviors of a single fluidized nanoparticle agglomerate with the fractal dimension between 2.2 and 2.8 and the Reynolds number between 0.1 and 400 have been investigated. Results showed that the drag coefficient is function of both the Reynolds number and the fractal dimension. The fractal dimension for fluidized agglomerates of SiO_2_ nanoparticles has been reported to be around 2.5 [34]. Based on the results, for a fluidized nanoparticle agglomerate with a particle size of 416 μm and a fractal dimension of 2.5 formed from the SiO_2_ nanoparticles with the primary particle size of 16 nm, the drag coefficient is expressed as follows:

(4) $$C_{d}=\frac{13.6}{Re_{s}}\left(1+0.1{R_{es}}^{0.874}\right).$$

**Figure 1:**
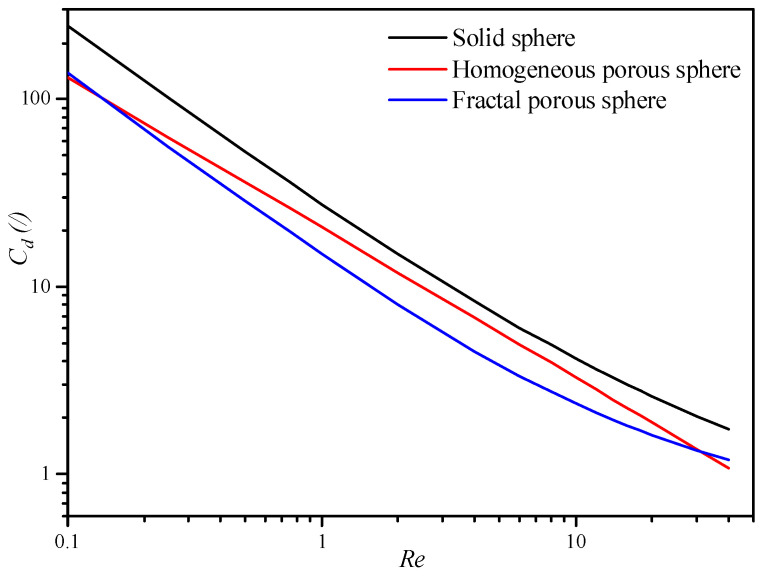
Variation in the drag coefficient with the Reynolds number.

In comparison with the drag force scale method based on the drag law of the solid sphere, the porous-structure-based drag laws mentioned above consider both the effects on the drag force of the pore structure of nanoparticle agglomerates and the hydrodynamic condition of gas flow. As shown in Figure 1, the drag coefficients calculated by the porous-structure-based drag laws are much lower than that by the drag law of the solid sphere. This will lead to a great reduction in the drag force on each single nanoparticle agglomerate. To access their applicability for modeling the fluidization of nanoparticle agglomerates under different flow regimes, drag laws of the homogeneous and fractal porous sphere are inserted into the framework of the Huilin–Gidaspow drag model in ANSYS Fluent 2020 R2 by user-defined functions in this work.

### 2.3. Model Set-Up and Parameters

**Figure 2:**
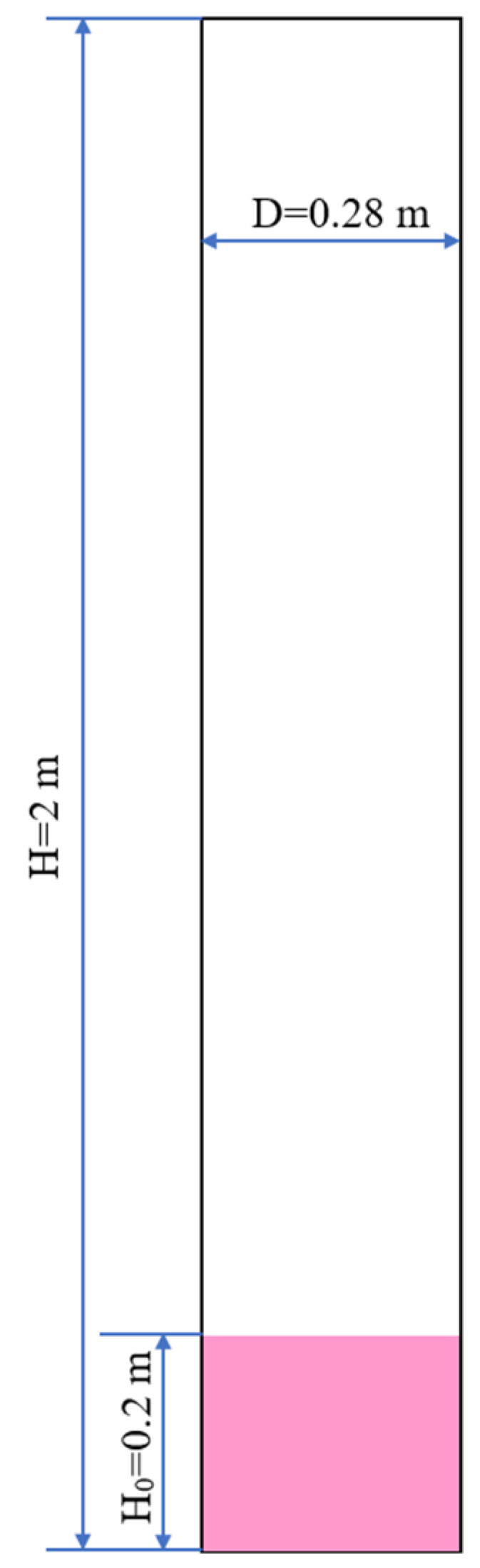
Simulated fluidized bed structure.

Huang et al. [35] used nanoparticles as fluidization materials and studied the solids mixing behavior in a fluidized bed experimentally. To compare the simulation results with their experimental results, the structure of the simulation is shown in Figure 2. To simplify the calculation, the model is set as a two-dimensional structure, and auxiliary structures in the experimental equipment are ignored. In this model, boundary conditions for velocity inlet, pressure outlet, and fixed walls are used. The gas phase is the air at normal temperature, and the gas velocity is in the range of 0.003~0.1 m/s. The gas flow outlet is set at the atmospheric pressure. The parameters selected from experimental data are listed in Table 1. Taking agglomerates of nano-sized SiO_2_ as bed material particles, the agglomerate size is 416 μm, the particle density is 2560 kg/m^3^, and the agglomerate density is set as 36.6 kg/m^3^, which is 1.15 times of the bulk density of 31.85 kg/m^3^ according to [35,36]. The initial packing height is 0.20 m.

### 2.4. Grid-Independent Test

**Figure 3:**
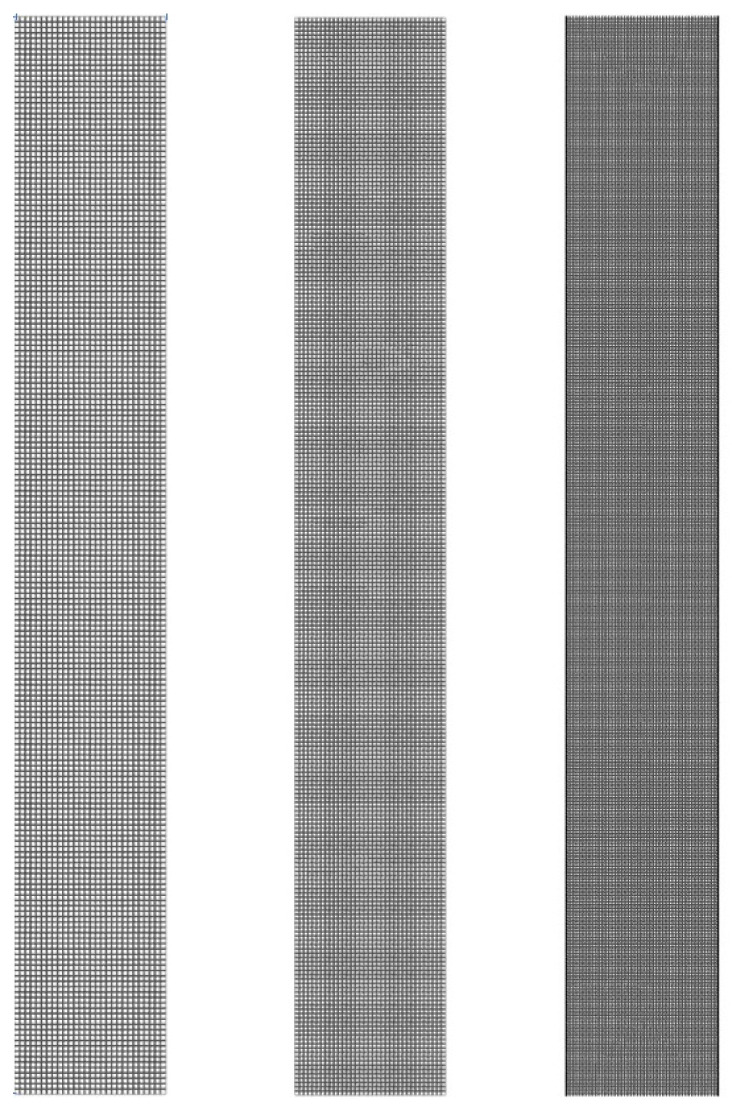
Mesh division from coarse to dense.

**Figure 4:**
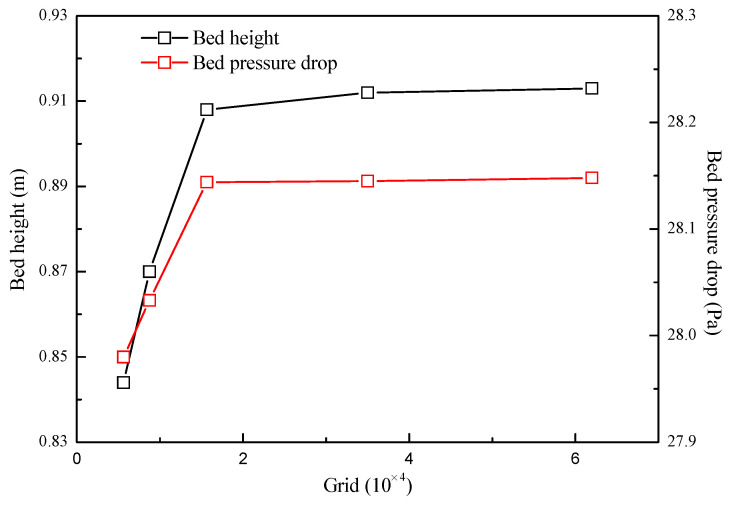
The grid-independent test.

To ensure a grid-independent solution and accurate resolution, a study has been performed with five sets of meshes for fluidization at 0.08 m/s. Three set of meshes from coarse to dense with the grid quantity of 8750, 15,651 and 35,000 are shown in Figure 3. It can be seen from Figure 4 that the difference in bed pressure drop and bed height between mesh 4 and 5 is less than 1.0%, which indicates that the solution based on mesh 4 is grid-independent. Considering the solution accuracy and time consumption, mesh 4 with a grid quantity of 35,000 was chosen for the simulation.

## 3. Results and Discussion

### 3.1. Bed Expansion Behaviors

**Figure 5:**
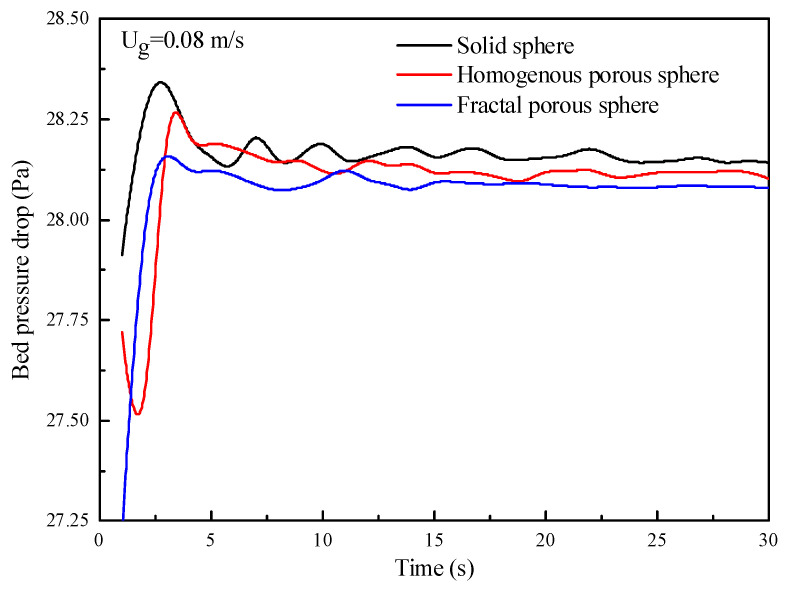
Evolution of the bed pressure drop with time.

Figure 5 shows the evolution of the bed pressure drop with time as obtained using the solid sphere drag law and the porous-structure-based drag laws. Initially, the three drag laws predict a sharp increase in bed pressure drop with time, and the bed pressure drops reach a peak value at about 3 s. The amount of pressure drops above about 28.1 Pa, which is the pressure drop that exceeds the weight of particles per unit area and has been referred to as “overpressure” as reported in the literature [37]. This overpressure is required at the initial fluidization stage to overcome the adhesion between the particles and the friction between the particles and the wall [37]. Subsequently, the fluidization steady state is reached after about 5 s, and a platform is then reached for the pressure drop, which fluctuates around a value of about 28.1 Pa corresponding to the weight of the bed divided by the cross-sectional area. Although the amplitude of the fluctuations decreases with time, the bubbling fluidization regime still causes considerable fluctuations for long simulation times. Therefore, the results are analyzed by averaging the simulated data from 20 to 30 s, which is sufficient to estimate flow behaviors of nanoparticle agglomerates in the fluidized bed.

The minimum fluidization velocity (*U_mf_*) refers to the critical velocity value at which a bed transitions from a stationary state to a fluidized state. In this work, it is obtained from the bed pressure drop curves and is defined as the superficial gas velocity from which the bed pressure drop reaches a plateau. The nanoparticle agglomerates all fluidize smoothly with no bubbles for three drag laws when the fluidization velocity is increased above the minimum fluidization velocity, that is, agglomerate particulate fluidization (APF). As the fluidization velocity is further increased, bubbling occurs in the bed, and agglomerate bubbling fluidization (ABF) is achieved. The minimum bubbling velocity (*U_mb_*) refers to the velocity value at which the first bubble in the fluidized bed appears, while visual observations of the appearance of the first bubble make it subjective [15]. In this work, *U_mb_* is determined by the sudden changes in granular temperature by two orders of magnitude. These determination criteria were introduced by Wang et al. [38] and verified by Motlagh et al. [15]. Table 2 shows the simulation and experimental data of the minimum fluidization velocity and minimum bubbling velocity. It can be seen from Table 2 that the minimum fluidization velocities obtained by the three drag laws are all close to the experimental data, but there are some differences in the minimum bubbling velocity among the three drag laws. Simulation with the fractal porous sphere drag law shows the largest minimum bubbling velocity, which means that the ABF is reached at a greater fluidization velocity. This leads to a wider APF range for fluidization simulated with the drag law of the fractal porous sphere. Yao et al. [39] has found that the fluidization of SiO_2_ nanoparticles shows a wider APF range than that of Geldart A particles. Our results indicate that this may be closely related to the lower drag force experienced by the highly porous nanoparticle agglomerates.

**Figure 6:**
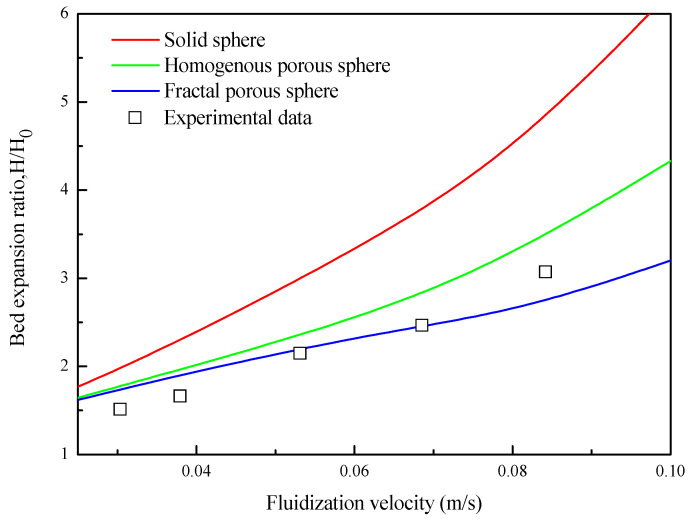
Bed expansion ratio of simulated results and experimental data from Ref. [35].

Figure 6 shows the bed expansion curves of nanoparticle agglomerates in a fluidized bed under different fluidization velocities with three different drag laws. It can be seen that when the fluidization velocity is between 0.023–0.038 m/s (APF), the differences in bed height with these three drag laws are small, and the simulated results are all very close to the experimental data. As the fluidization velocity is increased above 0.038 m/s (ABF), bubbles occur, more nanoparticle agglomerates are lifted up to the upper section of the bed, and the bed expansion ratio increases approximately linearly. At this time, the differences between the bed heights of different drag laws gradually increase, which may be related to the advanced appearance of bubbles of the fluidization simulated with the drag law of the solid sphere. The bed expansion of the solid sphere drag law is the highest among the three, and that of the drag law of the fractal porous sphere is the lowest and closest to the experimental data. This shows that the porous-structure-based drag laws make better predictions than those of the solid sphere in the ABF condition. This distinct comparison indicates that the pore structure of fluidized nanoparticle agglomerates inevitably affects their fluidization behavior. The drag force experienced by nanoparticle agglomerates is reduced, and the bed expansion is not as high at higher fluidization velocities. Therefore, pore structure effects on drag force need to be considered during simulation of fluidization of nanoparticle agglomerates.

### 3.2. Axial Solid Mixing Behavior

**Figure 7:**
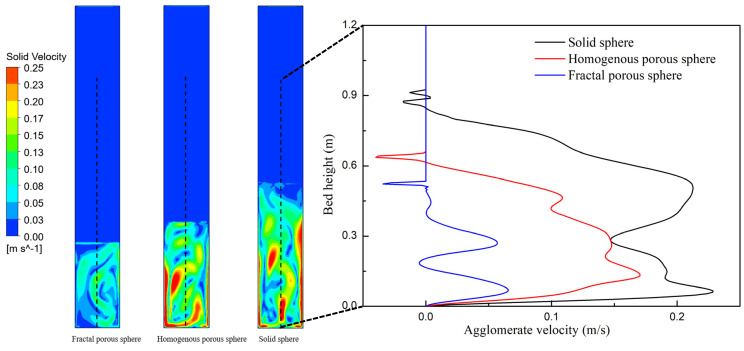
Instantaneous contour of the solid velocity at 25 s and time-averaged solid velocities of three drag laws.

**Figure 8:**
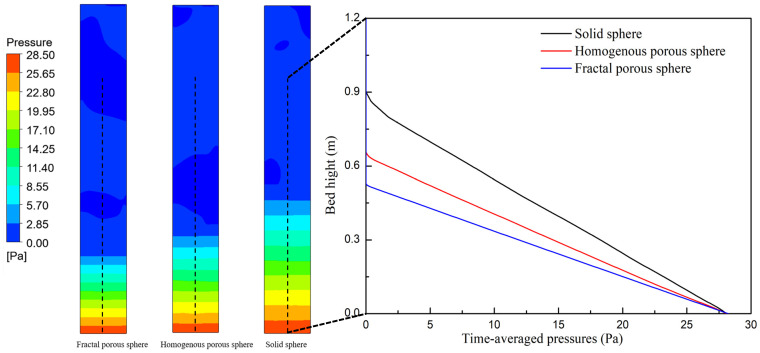
Instantaneous contour of the bed pressure at 25 s and time-averaged pressures of three drag laws.

Solid mixing behavior has an important effect on the heat and mass transfer in the fluidized reactor. Figure 7 shows the contours of the solid velocity and the variation in solid velocity with the bed height for three drag laws at a fluidization velocity of 0.08 m/s. It can be seen that fluidization simulated with the porous-structure-based drag laws shows weaker internal motion than the solid sphere drag law. At the same bed height, the solid velocities of the porous-structure-based drag laws are much lower than the solid sphere drag law. During the fluidization process, gas energy is converted into kinetic energy of solid phases. Perhaps due to the effect of pore structures, the momentum exchange between air and the nanoparticle agglomerates is much weaker than that of solid particles. Figure 8 shows the contours of the distribution of time-averaged pressure and the variation in axial pressure with the bed height for three drag laws at a fluidization velocity of 0.08 m/s. The axial time-averaged pressures of the three drag laws all decrease with the increase in the bed height. A dilute phase zone seems to appear for fluidization simulated with the solid sphere drag law.

**Figure 9:**
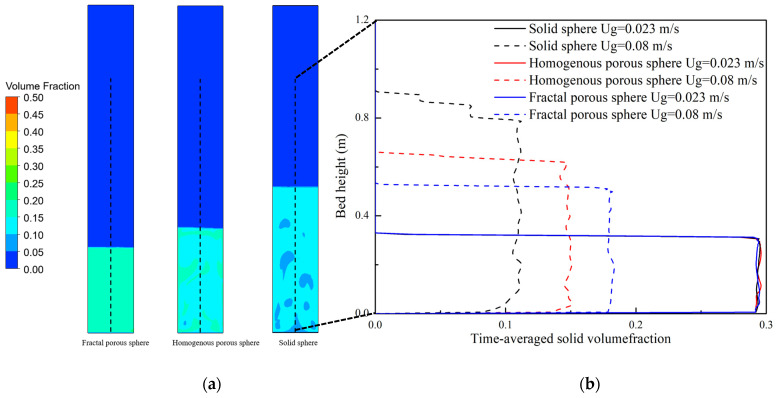
(**a**) Instantaneous contour of solid volume fraction at 25 s and (**b**) time-averaged solid volume fractions.

Figure 9a shows the contours of the solid volume fraction at a fluidization velocity of 0.08 m/s (ABF). The solid volume fraction of fluidization with the solid sphere drag law is the smallest, while that of the drag law of the fractal porous sphere is the largest. Large clear bubbles can be observed from fluidization with the solid sphere drag law. Figure 9b shows the variation in time-averaged solid volume fraction along the bed height for the three drag laws at a fluidization velocity of 0.023 m/s (APF) and 0.08 m/s (ABF). Overall, the agglomerate concentration decreases with increasing fluidization velocity. At the fluidization velocity of 0.023 m/s, the concentration distribution of the three drag laws is similar to each other. At the fluidization velocity of 0.08 m/s, nanoparticle agglomerates at the bottom of the bed are carried up by bubbles to the upper part of the bed. As a result, the agglomerate concentration at the bottom of bed decreases, while the agglomerate concentration in the upper section of bed increases.

### 3.3. Radial Solid Mixing Behavior

**Figure 10:**
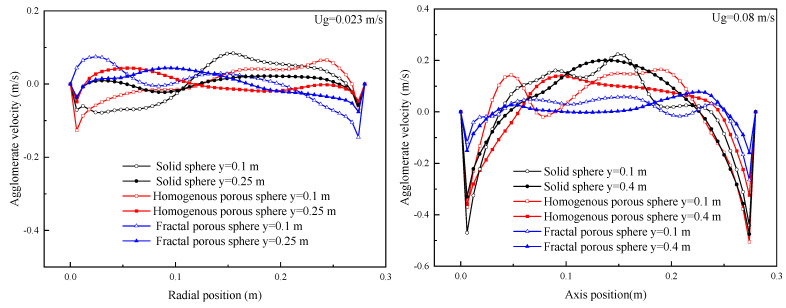
Radial distribution of time-averaged agglomerate velocity.

Figure 10 shows the radial distribution curves of agglomerate velocity at different heights for different drag laws. When the fluidization velocity is 0.023 m/s, the fluidization of nanoparticle agglomerates is uniformly distributed in the bed for the three drag laws, and the agglomerate velocity is not sensitive to the bed height. These are consistent with APF characteristics. When the fluidization velocity is increased to 0.08 m/s (ABF), it can be seen that the radial distribution of agglomerate velocity for three drag laws has the same characteristics. The agglomerate velocities are all parabolic-like distributed in the bed. The agglomerate velocities reach their maxima at the bed center and decrease toward the bed walls. The positive agglomerate velocity means the nanoparticle agglomerates move upward, and the negative agglomerate velocity means that the nanoparticle agglomerates move downward. Upward flows of nanoparticle agglomerates at the center and downward flows near the bed walls indicate that the nanoparticle agglomerates are circulating in the bed, and a core-annular flow structure is formed. The agglomerate velocity differences between the center and annulus of the bed simulated with the porous-structure-based drag laws, especially the drag law of fractal porous sphere, are smaller than that of the drag law of solid sphere. This indicates that core-annular flow is weaker for fluidization with the porous-structure-based drag laws. Comparison between the two figures in Figure 10 shows that the agglomerate velocity is increased in the center of the bed with increasing fluidization velocity. The agglomerate velocity is also increased in the annulus to balance the solid mass flux between the center and annulus. Hence, large fluidization velocity will increase the agglomerate mixing in the bed.

**Figure 11:**
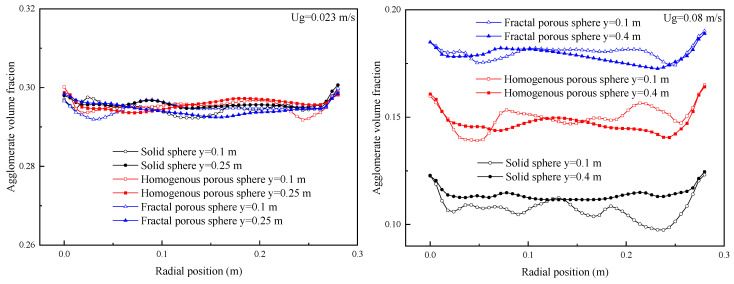
Radial distribution of time-averaged agglomerate concentration.

Figure 11 shows the radial distribution curves of the time-averaged agglomerate concentration of the three drag laws at different heights. When the fluidization velocity is 0.023 m/s, the nanoparticle agglomerates are almost uniformly distributed in the bed for all of the three drag laws with a slightly higher agglomerate concentration near bed walls. The agglomerate concentration is not sensitive to the bed height. When the fluidization velocity is increased to 0.08 m/s (ABF), a core-annular flow structure is observed as evident for the time-averaged agglomerate concentration distributions. The solid volume fraction is low in the center while being high in the annular zone as expected. The agglomerate concentration decreases in both the center and in the annulus with increasing fluidization velocity. The high fluidization velocity will dilute the agglomerate concentration in the bed. It should be noted that the differences in agglomerate concentration predicted by the three drag laws are obvious at this time. The agglomerate concentration is increased gradually and significantly for fluidization simulated with the drag law of the solid sphere and the homogeneous and fractal porous sphere. 

### 3.4. Agglomerate Dispersion Coefficient

To further quantitatively analyze the applicability of the porous-structure-based drag laws for modeling the fluidization of nanoparticle agglomerates under different velocities, the solid mixing behavior is analyzed. It was described by a two-dimensional model which was validated as a successful model in previous research [40,41]. In this model, the agglomerate dispersion is characterized by an axial dispersion coefficient $D_{a}$ and a radial dispersion coefficient $D_{r}$. Based on the simulation results from the two-fluid model, tracing particles were injected into the bed to track the solid phase movement of stable simulation after 30 s, and the average solid dispersion coefficients were computed according to these trajectories as follows:

(5) $$Y=y\left(t\right)-y_{0},$$

(6) $$\bar{Y}=\frac{1}{N}\sum_{n=1}^{N}y_{n},$$

(7) $${\bar{Y}}^{2}=\frac{1}{N}\sum_{n=1}^{N}{\left(Y_{i}-\bar{Y}\right)}^{2},$$

(8) $$D_{a}=\frac{1}{2}\frac{d{\bar{Y}}^{2}}{dt},$$

where  Y is the instantaneous axial displacement, $\bar{Y}$ is the averaged axial displacement of tracing particles, and ${\bar{Y}}^{2}$ is the averaged square of axial displacement.

**Figure 12:**
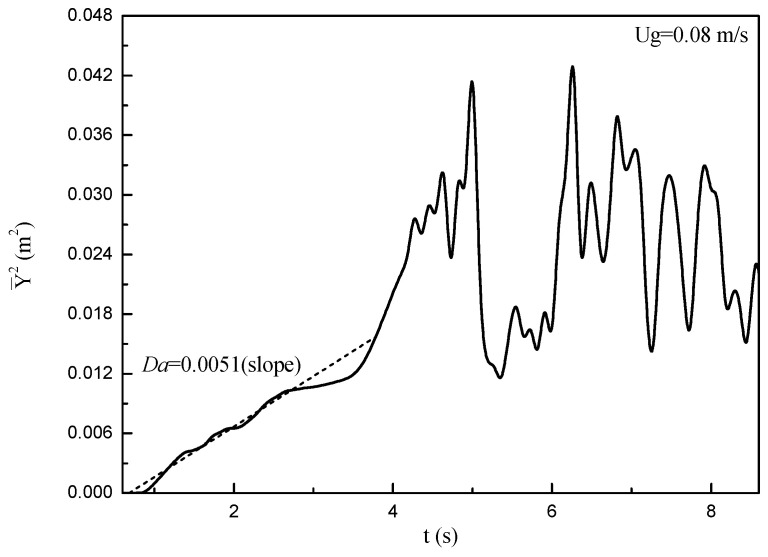
Averaged square of axial displacement with time.

Figure 12 shows the evolution of ${\bar{Y}}^{2}$ with time after the injection of tracing particles. It can be seen that ${\bar{Y}}^{2}$ increases linearly with time until it reaches almost a constant value and oscillates around it, which shows that tracing particles are well dispersed in the bed. The axial dispersion coefficient $D_{a}$ is obtained from regressing a line on the linear part of the curve, and it is equal to half of the slope of the linear part of the curve. The values of $D_{r}$ can be obtained by the same method.

**Figure 13:**
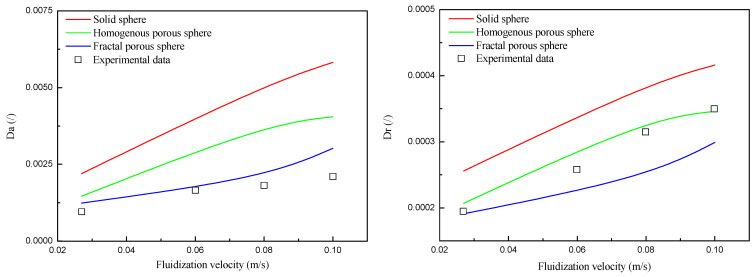
$D_{a}$ and $D_{r}$ of simulation results and experimental data from ref. [35].

Figure 13 shows values of $D_{a}$ and $D_{r}$ for fluidization simulated with the three drag laws under different fluidization velocities. $D_{a}$ and $D_{r}$ both increase monotonically with the increase of fluidization velocity, indicating that increasing fluidization velocity is beneficial for promoting the solid mixing behavior. This is consistent with the report by Farahani et al. [42], in which they found that the solid diffusivities increase with the superficial velocity. Bubbles are mainly responsible for solid mixing behavior in fluidized beds. The axial mixing behavior of nanoparticle agglomerates is mainly influenced by the rising velocity of bubbles and the solid circulation in the regions surrounding the rising bubbles. The radial mixing of nanoparticle agglomerates is closely related to the lateral motion of bubbles and lateral dispersion of particles in the bubble’s wake [42]. As the fluidization velocity is increased, the diameter and rising velocity of bubbles are both increased, and their ability to carry agglomerates up with them is then increased. Meanwhile, the interaction of neighboring bubbles is intensified, and the coalescence of neighboring bubbles and the eruption of the bubble occur more frequently. The above behaviors lead to an increase in the $D_{a}$ and $D_{r}$ with the superficial velocity.

In addition, it can be seen that the $D_{a}$ values are within the range of 0.001~0.006 and the $D_{r}$ values are within the range of 0.0002~0.0004 for different drag laws. These values are much smaller compared to those of other Geldart groups of particles, indicating that the mixing behavior in the fluidized bed of nanoparticle agglomerates is much less intense than other Geldart groups of particles. This should be mostly related to the low density and small size of the nanoparticle agglomerates. The values of $D_{a}$ and $D_{r}$ for fluidization simulated with the solid sphere drag law are larger than the others, which means that the solid mixing behaviors for fluidization simulated with the solid sphere drag law are much stronger. This should be related to the phenomenon that more and larger bubbles occur in the fluidized bed simulated with the drag law of the solid sphere shown in Figure 9. It should also be noticed that the simulation results with porous-structure-based drag laws are in good agreement with the experimental data. This indicates that, without considering the effect of pore structure on the two phases, the simulation results of flow behaviors could deviate from the experimental data.

## 4. Conclusions

In the present study, numerical simulations were performed to evaluate the applicability of porous-structure-based drag laws for a two-dimensional nanoparticle agglomerate fluidized bed from particulate to bubbling fluidization state. A Eulerian–Eulerian two-fluid model was used by incorporating different drag laws of the homogenous and fractal porous spheres by considering the effect of the highly porous structure for the solid phase. Validity of the present simulation was assessed through comparisons of simulation results and experimental data of fluidized bed hydrodynamics. The simulation was also used to investigate axial and radial solid mixing behaviors under particulate and bubbling fluidization conditions. The simulation results reveal that the choice of drag laws can significantly affect the fluidized bed hydrodynamics. Simulated results of bed expansion ratio, the minimum fluidization and bubbling velocities, and agglomerate dispersion coefficient predicted by the porous-structure-based drag laws are in good agreement with experimental data. In comparison, the solid sphere drag law makes a good prediction in the particulate fluidization condition and an unsatisfying prediction in the bubbling fluidization condition. This indicates that the pore structure has a great impact on the gas–solid flow, and the porous-structure-based drag laws are suitable for describing flows in nanoparticle agglomerate fluidized beds. The current simulation is assuming nanoparticle agglomerates are average-sized and spherical. We plan to make a further study to consider the influence of agglomerate size and shape.

## Figures and Tables

**Table 1:** Parameters used for simulations of experiments from Ref. [35].

Property	Symbol (Unit)	Value
Nanoparticle diameter	d_p_ (nm)	16
Agglomerate diameter	d_a_ (μm)	416
Particle density	ρ_p_ (kg/m^3^)	2560
Agglomerate density	ρ_b_ (kg/m^3^)	36.6
Gas viscosity	µ_f_ (Pa·s)	1.81 × 10^−5^
Gas density	ρ_f_ (kg/m^3^)	1.205
Initial packing height	H_0_ (m)	0.2
Maximum packing limit	α_p,max_	0.67

**Table 2:** The minimum fluidization and bubbling velocity.

	Solid Sphere	Homogenous Porous Sphere	Fractal Porous Sphere	Experimental [35]
$U_{mf}$ (m/s)	0.007	0.007	0.007	0.007
$U_{mb}$ (m/s)	0.007	0.027	0.042	0.038

## Data Availability

The data presented in this study are available on request from the corresponding author.

## Supplementary Materials

The following supporting information can be downloaded at: https://www.mdpi.com/article/10.3390/nano14121057/s1, Section S1: Governing equations for gas/solid flows; Section S2: Constitutive equations of gas-solid flow; Section S3: The drag model used in this work. Refs. [43,44] are cited in Supplementary Materials.
