# Supplementary material for: Flow Behavior of Nanoparticle Agglomerates in a Fluidized Bed Simulated with Porous-Structure-Based Drag Laws
Source: Nanomaterials (Basel). 2024 Jun 19;14(12):1057. doi: 10.3390/nano14121057 (PMC11207026; doi:10.3390/nano14121057)
Supplement: Supplementary file 1 [file nanomaterials-14-01057-s001.zip › nanomaterials-3011239-supplementary.pdf]

nanomaterials

Supplementary Materials

Flow behavior of nanoparticle agglomerates in a fluidized bed simulated with porous-structure-based drag laws

Shaowei Wang[1,*], Xiaobing Hu[1], Niannian Liu[2] and Huanpeng Liu[3]

[1] *Energy and Power Engineering Institute, Henan University of Science and Technology, Luoyang, Henan 471003, PR China*
[2] *Department of Engineering Mathematics, University of Bristol, Bristol, United Kingdom*
[3] *School of Energy Science and Engineering, Harbin Institute of Technology, Harbin, Heilongjiang 150006, PR China*
* Correspondence: 9906235@haust.edu.cn; Tel.: 0086-379-64231480

Section S1. Governing equations for gas/solid flows.

Mass conservation for phase i (i = *g* for gas and *s* for solid):

$$\frac{\partial(\varepsilon_i\rho_i)}{\partial t} + \nabla\cdot(\varepsilon_i\rho_i\vec{v}_i) = 0$$

Linear momentum balance for gas and solid phases:

$$\frac{\partial(\varepsilon_g\rho_g\vec{v}_g)}{\partial t} + \nabla\cdot(\varepsilon_g\rho_g\vec{v}_g) = -\varepsilon_g\nabla p_g + \nabla\cdot\bar{\bar{\tau}}_g + \beta_{gs}|\vec{v}_s - \vec{v}_g| + \varepsilon_g\rho_g\vec{g}$$

$$\frac{\partial(\varepsilon_s\rho_s\vec{v}_s)}{\partial t} + \nabla\cdot(\varepsilon_s\rho_s\vec{v}_s) = -\varepsilon_s\nabla p_s + \nabla\cdot\bar{\bar{\tau}}_s + \beta_{gs}|\vec{v}_s - \vec{v}_g| + \varepsilon_s\rho_s\vec{g}$$

Transport equation for the solid phase granular temperature:

$$\frac{3}{2}\left[\frac{\partial}{\partial t}(\varepsilon_s\rho_s\Theta_s) + \nabla\cdot(\varepsilon_s\rho_s\vec{v}_s\Theta_s)\right] = \left(-p_s\bar{\bar{I}} + \bar{\bar{\tau}}_s\right)\cdot\nabla\vec{v}_s + \nabla\left(k_{\Theta_s}\nabla\Theta_s\right) - \gamma_{\Theta_s} + \Phi_{gs}$$

Section S2. Constitutive equations of gas-solid flow.

Gas stress tensor:

$$\bar{\bar{\tau}}_g = \varepsilon_g \mu_g \left[\nabla v_g + \nabla v_g^T\right] - \frac{2}{3}\varepsilon_g \mu_g \nabla \cdot v_g \vec{I}$$

Solid stress tensor:

$$\bar{\bar{\tau}}_s = \mu_s \left[\nabla v_s + \nabla v_g^T\right] - \frac{2}{3}\mu_s \nabla \cdot v_s \vec{I}$$

where solid shear viscosity is:

$$\mu_s = \mu_{s,col} + \mu_{s,kin} + \mu_{s,fr}$$

The collisional contribution to shear viscosity is:

$$\mu_{s,col} = \frac{4}{5}\varepsilon_s \rho_s g_{0,ss}(1+e_{ss})\sqrt{\frac{\Theta_s}{\pi}}$$

The kinetic contribution to shear viscosity:

$$\mu_{s,kin} = \frac{10 d_s \rho_s \sqrt{\Theta_s \pi}}{96 \varepsilon_s g_{0,ss}(1+e_{ss})}[1 + \frac{4}{5}\varepsilon_s g_{0,ss}(1+e_{ss})]^2$$

The frictional contribution to shear viscosity [1]:

$$\mu_{s,fr} = p_f \sin(\varphi)/2\sqrt{I_{2D}}$$

Solid phase bulk viscosity:

$$\lambda_s = \frac{4}{3}\varepsilon_s d_s \rho_s g_{0,ss}(1+e_{ss})\ {}^{2}\sqrt{\frac{\Theta_s}{\pi}}$$

Collision dissipation energy [2]:

$$\gamma_\Theta = \frac{12(1-e_{ss}^2)g_{0,ss}}{d_s\sqrt{\pi}}\rho_s \varepsilon_s^2 \Theta_s^{3/2}$$

Solids pressure:

$$p_s = \varepsilon_s \rho_s \Theta_s + 2\rho_s(1+e_{ss})\varepsilon_s^2 g_{0,ss}$$

Thermal energy diffusion coefficient:

$$k_\Theta = \frac{25 d_s \rho_s \sqrt{\Theta_s \pi}}{64 g_{0,ss}(1+e_{ss})}[1 + \frac{6}{5}\varepsilon_s g_{0,ss}(1+e_{ss})]^2 + 2\rho_s \varepsilon_s^2 d_s g_{0,ss}(1+e_{ss})\sqrt{\frac{\Theta_s}{\pi}}$$

Radial distribution function:

$$g_{0,ss} = [1 - (\frac{\varepsilon_s}{\varepsilon_{s,max}})^{1/3}]^{-1}$$

Dissipation of granular temperature by gas damping:

$$\Phi_{gs} = -3\beta\Theta_s$$

Section S3. The drag model used in this work.

Huilin-gidaspow drag model [3]:

Huilin-gidaspow drag model is a combination of the previous drag models proposed by Wen and Yu [4] and Ergun [5]. It introduces a transition function to smoothly switch the drag coefficients at the bed porosity of 0.8. The pressure drop due to friction between gas and particles is described by the Ergun equation [5] as the bed porosity is less than 0.8 and Wen and Yu equation as the bed porosity is larger than 0.8.

$$\beta_1 = 150\frac{\varepsilon_s(1-\varepsilon_g)\mu_g}{\varepsilon_g d_a^2} + 1.75\frac{\rho_g \varepsilon_s|\vec{v}_s-\vec{v}_g|}{d_a} \quad (\varepsilon_g \leq 0.8)$$

$$\beta_2 = \frac{3}{4}C_d\frac{\varepsilon_s\varepsilon_g\rho_g|\vec{v}_s-\vec{v}_g|}{d_a}\varepsilon_g^{-2.65} \quad (\varepsilon_g > 0.8)$$

$$\beta_{gs} = \beta_1 \quad (\varepsilon_g < 0.8)$$

$$\beta_{gs} = \varphi\beta_1 + (1-\varphi)\beta_2 \quad (\varepsilon_g > 0.8)$$

$$\varphi = \frac{1}{2} + \frac{\arctan\left(262.5(\varepsilon_s - 0.2)\right)}{\pi}$$

where drag coefficient is:

$$C_d = \frac{24}{Re_s}\left[1 + 0.15(Re_s)^{0.687}\right] \quad (Re_s < 1000)$$

$$Re_s = \frac{\rho_g d_a\left|\vec{v}_s - \vec{v}_g\right|}{\mu_g}$$

References

1. Johnson P C, Jackson R. Frictional-collisional constitutive relations for granular materials, with application to plane shearing. *Journal of Fluid Mechanics*, 1987, 176.
2. Gidaspow D. Multiphase Flow and Fluidization: Continuum and Kinetic Theory Description. *Journal of NonNewtonian Fluid Mechanics*, 1994, 55(2):207-208.
3. Huilin Lu, Gidaspow D, Bouillard J, et al. Hydrodynamic simulation of gas-solid flow in a riser using kinetic theory of granular flow. *Chemical Engineering Journal*, 2003, 95(1-3):1-13.
4. Wen C, Yu Y. A Generalised Method for Predicting Minimum Fluidization Velocity. *AICHE Journal*.1966, 12.
5. Ergun S. Fluid Flow through Packed Columns. *Chemical Engineering Progress*, 1952, 48:89-94.
